# “INCORPORATE INTO MY DAILY LIFE”: RESPONSES TO MITOFIT INTERVENTION VIDEOS ON MITOCHONDRIAL FITNESS

**DOI:** 10.1093/geroni/igae098.2439

**Published:** 2024-12-31

**Authors:** Jeffrey Boon, Brandon Grubbs, Maulik Patel, John Dunavan, Kelly Knickerbocker, Cathy Maxwell

**Affiliations:** Vanderbilt University Medical Center, Nashville, Tennessee, United States; Middle Tennessee State University, Murfreesboro, Tennessee, United States; Vanderbilt University, Nashville, Tennessee, United States; Peer-Tree, Franklin, Tennessee, United States; Vanderbilt University, Nashville, Tennessee, United States; University of Utah College of Nursing, Salt Lake City, Utah, United States

## Abstract

Mitochondrial fitness is a term that reflects the ability of mitochondria to adapt/maintain optimal performance. As a key predictor of age-related decline, mitochondrial fitness is a target for interventions in healthy aging. MitoFit is a novel instructional communication intervention designed for improving physical activity in persons aged 50 and over. We aimed to determine participants’ responses to the MitoFit educational videos and clarify factors that contributed to reactions.

**Methods:**

Focus groups with community-dwelling adults (aged 50+) viewed six literacy-friendly MitoFit videos two at a time followed by a semi-structured group interviews to elicit verbal feedback. Interviews were audio-recorded and transcribed verbatim. Transcribed data were independently coded by three researchers, followed by a consensus process to identify themes, codes and subcodes.

**Results:**

101 adults (mean age: 67.8 years (8.9); 75.0% female) participated in 15 focus groups at six sites. A conceptual model emerged, centered around the concept of cognitive restructuring (mental adjustments to prior or unhelpful beliefs) related to understanding about exercise. Contributing themes included: a) initial reactions (codes: impactful, motivating, fascinating), b) biological content (codes: this is biology, battery analogy, energetics), and c) personal integration (codes: prompts reflection, unaware, leads to more questions). The theme of key take-aways included codes: a) have to do it (subcodes: consistency, find what works, find what I like, making a plan), b) hope (subcode: not too late), c) reversible, and d) sense of agency (subcode: doing it for me).

**Conclusion:**

MitoFit video content provoked positively strong reactions that may contribute to self-efficacious lifestyle change.

